# X-ray structure determination at low resolution

**DOI:** 10.1107/S0907444908043795

**Published:** 2009-01-20

**Authors:** Axel T. Brunger, Byron DeLaBarre, Jason M. Davies, William I. Weis

**Affiliations:** a Howard Hughes Medical Institute, Stanford University, USA; b Department of Molecular and Cellular Physiology, Stanford University, USA; c Department of Neurology and Neurological Sciences, Stanford University, USA; d Department of Structural Biology, Stanford University, USA; e Department of Photon Science, Stanford University, USA

**Keywords:** refinement, low resolution, structure validation, ATPases, p97/VCP

## Abstract

As an example of structure determination in the 3.5–4.5 Å resolution range, crystal structures of the ATPase p97/VCP, consisting of an N-terminal domain followed by a tandem pair of ATPase domains (D1 and D2), are discussed. The structures were originally solved by molecular replacement with the high-resolution structure of the N-D1 fragment of p97/VCP, whereas the D2 domain was manually built using its homology to the D1 domain as a guide. The structure of the D2 domain alone was subsequently solved at 3 Å resolution. The refined model of D2 and the high-resolution structure of the N-D1 fragment were then used as starting models for re-refinement against the low-resolution diffraction data for full-length p97. The re-refined full-length models showed significant improvement in both secondary structure and *R* values. The free *R* values dropped by as much as 5% compared with the original structure refinements, indicating that refinement is meaningful at low resolution and that there is information in the diffraction data even at ∼4 Å resolution that objectively assesses the quality of the model. It is concluded that *de novo* model building is problematic at low resolution and refinement should start from high-resolution crystal structures whenever possible.

## Abstract 2

Refinement is meaningful even at 4 Å or lower, but with present methodologies it should start from high-resolution crystal structures whenever possible.

## 1. Introduction

As more and more challenging and complex systems are being studied, X-ray crystallography is increasingly hampered by weakly diffracting crystals. Intrinsic disorder or flexibility of large macromolecular assemblies prevents the growth of sufficiently large regular lattices, which results in weak diffraction and/or extremely small crystals. It is therefore important to develop new experimental and computational methods that achieve the maximum possible information from such low-resolution diffraction data (limiting resolution worse than 3.5 Å).

In macromolecular complexes, the structures of some of the components may be known at high resolution, while those of others are unknown. Solving such complexes should be possible as the determinancy point, that is the Bragg spacing limit at which the number of independent diffraction intensities equals the number of flexible torsion angles, is generally in the range between 4.9 and 6.4 Å; for example, for a crystal structure with 50% solvent content the determinancy point is 5.4 Å (Hendrickson, 2009 ▶). Thus, it should in principle be possible to determine all flexible torsion angles of a macromolecular crystal structure even at low resolution. Unfortunately, for a low-resolution diffraction data set, current methods require a high-resolution starting structure for the entire macromolecular structure (Davies *et al.*, 2008 ▶). In this paper, we describe our experiences with the structure solution and refinement of a difficult low-resolution crystal structure, the ATPase p97/VCP.

The ATPase p97/VCP consists of an N-terminal domain followed by a tandem pair of ATPase domains (D1 and D2). We obtained low-resolution diffraction data sets of p97/VCP in three nucleotide states (ATP at 3.5 Å resolution, ATP·AlF*_x_* at 4.5 Å resolution and ADP at 4.25 Å resolution; DeLaBarre & Brunger, 2003 ▶, 2005 ▶) and employed molecular replacement using the known structure of the N-D1 fragment (PDB code 1e32) as the search model; the structure of the isolated D2 domain was unknown at the time. Combined with selenomethionine (SeMet) multi-wavelength anomalous dispersion (MAD) phasing of the entire molecule, we traced the backbone of the D2 domain and the linker connecting it to the N-­D1 fragment. *R*_free_ values of below 32% were achieved upon refinement that included experimental phase information using the MLHL maximum-likelihood target function together with judicious use of noncrystallographic symmetry (NCS) and secondary-structure restraints. Significant conformational changes could be discerned by comparison of the structures solved in the three nucleotide states. However, the secondary-structural elements of the D2 domain showed important deviations from ‘typical’ ATPase structures solved at high resolution and the definition of secondary structure was poor throughout the model. The electron-density maps did not provide any clues to how to improve the model. We refer to these structures as the ‘original’ p97/VCP models (‘obsolete’ PDB codes 1yqo, 1yqi and 1ypw).

Subsequently, we solved the D2 domain alone at 3 Å resolution in a crystal form with 14-fold noncrystallographic symmetry, which produced a high-quality structure for this domain (PDB code 3cf0; Davies *et al.*, 2008 ▶). The revised model of D2, in conjunction with the high-resolution structure of the N-D1 fragment, was used as a starting model for re-refinement against the low-resolution structure factors for full-length p97/VCP in the three nucleotide states. The revised full-length models showed significant improvement in both model geometry and *R* values compared with the original structures (Davies *et al.*, 2008 ▶). We refer to those models as the re-refined p97/VCP structures (PDB codes 3cf1, 3cf2 and 3cf3).

The p97/VCP example illustrates that it is possible to refine low-resolution crystal structures to relatively high quality if the structures of all components or domains are available at high resolution. We first summarize a number of special considerations for low-resolution refinements and then discuss our experiences with p97/VCP. A list of general suggestions and requirements for low-resolution refinements is provided in Table 1 ▶.

## 2. Special methods for low-resolution refinement

### 2.1. Bulk-solvent modeling and anisotropic scaling

The correct modeling of the disordered solvent in the crystal lattice is an important part of macromolecular structure refinement and it becomes especially important for structures determined at low resolution. The structure factor *F*_calc_ of a macromolecular crystal structure is 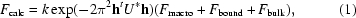
where the structure factor *F*_macro_ is obtained from the atomic model of the macromolecule, *F*_bound_ is computed from all bound water molecules, *F*_bulk_ is obtained from an appropriate model for disordered solvent, **h** is a column vector with the Miller indices of a Bragg reflection and *t* denotes its transpose (*i.e.* a row vector), *k* is a scale factor and the symmetric second rank tensor *U* describes overall mean-square displacements of the crystal lattice (dimensionless anisotropic mean-square displacements; ADPs). The isotropic component of the ADPs is usually separated from *U* and applied directly to *F*_macro_, *F*_bound_ and *F*_bulk_. To do this, the *U* tensor is converted into Cartesian coordinate space *U*_cart_ (Grosse-Kunstleve & Adams, 2002 ▶). One third of its trace {*i.e.* [*U*_cart_(11) + *U*_cart_(22) + *U*_cart_(33)]/3} is the isotropic thermal factor contribution.

To compute *F*_bulk_, one approach is to create a mask in order to distinguish between macromolecular and solvent regions (Brunger, 2007 ▶; Jiang & Brünger, 1994 ▶; Phillips, 1980 ▶). All grid points of the mask are initially set to 1. Grid points of the mask within a distance of *r*_*i*_ around any atom *i* of the atomic model and its symmetry mates are then set to 0. The atomic model includes the macromolecule and any bound water molecules or ligands. *r*_*i*_ is defined as the sum of the van der Waals radius *r*_vdw_ of atom *i* and the probe radius *r*_probe_. All grid points of the mask marked 0 are tested to see if they fall within a distance *r*_shrink_ from a grid point set to 1. If this is the case, the tested grid point is set to 1. This procedure effectively ‘shrinks’ the accessible surface area. For refinements up to around 3 Å resolution *R*_probe_ = *R*_shrink_ = 1 is the optimum choice (Jiang & Brünger, 1994 ▶). However, for low-resolution refinement the optimum values can differ from one (DeLaBarre & Brunger, 2003 ▶, 2005 ▶). The grid points of the mask marked 1 comprise the solvent regions, whereas those marked 0 are associated with the atomic model and its symmetry mates.

The structure factor of the solvent *F*_bulk_ is then simply computed by Fourier transformation of the mask. In order to blur the sharp boundary between macromolecule and solvent as imposed by the mask, resolution-dependent scaling in reciprocal space is applied using an isotropic ‘thermal’ factor *B*_sol_,
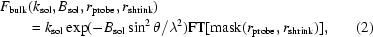
where FT denotes the three-dimensional Fourier transformation and *k*_sol_ is a scale factor that defines the mean electron density in the solvent region. For a well behaved aqueous solvent model *k*_sol_ is generally in the range 0.3–0.4 e Å^−3^ and *B*_sol_ is close (within a factor of two) to the average thermal factor of the macromolecular model (P. D. Adams, personal communication).

The optimum solvent model is obtained by minimizing the expression
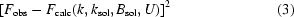
as a function of the anisotropic thermal factor *U*, the scale factor *k* and the bulk-solvent parameters *k*_sol_ and *B*_sol_, where *F*_obs_ is the observed structure factor. A straightforward application of least-squares optimization to determine the minimum of this expression results in numerical instabilities for structures determined at lower than 3 Å resolution. To avoid this problem, grid-search optimization has been used (Afonine *et al.*, 2005 ▶; Brunger, 2007 ▶). An implementation in *CNS* uses a one-dimensional grid search for *k*_sol_ and *R*_probe_ = *R*_shrink_ while letting *B*_sol_ and the other adjustable parameters be determined by least-squares optimization for each selected value of *k*_sol_ (Brunger, 2007 ▶). Another implementation in *Phenix* utilizes a two-dimensional grid search with both *k*_sol_ and* B*_sol_ and fixed *R*_probe_ = *R*_shrink_ = 1 (Adams *et al.*, 2002 ▶). Both implementations (which are available in the latest versions of *CNS* and *Phenix*) are robust over a wide range of minimum Bragg spacings of the diffraction data, especially at low resolution.

### 2.2. Treatment of weak intensities

With the emergence of maximum-likelihood-based refinement methods (Adams *et al.*, 1997 ▶; Pannu *et al.*, 1998 ▶) it is possible to include all weak diffraction data in refinement. Clearly, this is especially important when analyzing crystals that only diffract to low resolution. Weak reflections with large experimental error estimates are automatically down-weighted in the likelihood-based target function. R. Read suggested using the resolution-dependence of σ_A_ as a guide to determine the effective resolution limit (Ling *et al.*, 1998 ▶). We applied this approach to set the resolution limit for p97/VCP in complex with ADP; the suggested resolution limit corresponded to a conventional *I*/σ(*I*) cutoff of 1.2. For the ADP·AlF_*x*_ and AMP-PNP-ligated structures this approach resulted in *I*/σ(*I*) cutoffs as low as 0.8 (DeLaBarre & Brunger, 2005 ▶). We observed slight improvements in electron-density maps upon the inclusion of all weak diffraction data in the refinement and map calculations. A possible generalization of this approach would be to take into account anisotropic diffraction since this is commonplace for crystals of large macromolecular assemblies.

### 2.3. Thermal factor sharpening of electron-density maps

Thermal (‘*B*’) factor sharpening is a useful tool for the enhancement of low-resolution electron-density maps (Bass *et al.*, 2002 ▶; DeLaBarre & Brunger, 2003 ▶, 2005 ▶, 2006 ▶). Thermal factor sharpening entails the use of a negative *B*_sharp_ value in a resolution-dependent weighting scheme applied to a particular electron-density map:
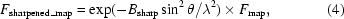
where *F*_map_ is the structure factor of the particular electron-density map, *F*_sharpened_map_ is the structure factor of the sharpened map, θ is the reflecting angle and λ is the wavelength of the X-ray radiation. A reasonable choice for *B*_sharp_ is the negative Wilson *B* value of the diffraction data. Since the customary procedure to obtain the Wilson *B* value requires high-resolution diffraction data, a maximum-likelihood-based method should be used for low-resolution data sets as described by Popov & Bourenkov (2003 ▶) and implemented in *Phenix* (Adams *et al.*, 2002 ▶).

Applying a negative *B*_sharp_ value effectively up-weights higher resolution terms. The result of this weighting scheme is increased detail for higher resolution features such as side-chain conformations. However, the cost of the increased detail can be increased noise throughout the electron-density map. Thus, thermal factor sharpening is a density-modification technique that is only as good as the diffraction data and phases that are available and therefore the original un­weighted electron-density maps should always be considered. *B*-factor sharpening provided some utility for the refinement of the original p97/VCP models, but it proved even more useful for the re-refined structures owing to improved model phase accuracy (Davies *et al.*, 2008 ▶).

## 3. Results

### 3.1. Original p97/VCP structures

We originally solved and refined the full-length p97/VCP structures without the availability of a high-resolution structure of the D2 domain. Initial phases for the crystal structure of the entire p97/VCP hexamer were obtained by molecular replacement with the N-D1 fragment (PDB code 1e32; Zhang *et al.*, 2000 ▶) using a 4.7 Å data set of the ADP·AlF_*x*_ nucleotide state (DeLaBarre & Brunger, 2003 ▶).

The resulting difference electron-density map showed many features that were consistent with the presence of a folded D2 domain. However, owing to the poor quality of the map, the D2 domain could not be traced. We therefore resorted to measurement of experimental phase information by SeMet MAD. The phases from a molecular-replacement solution with the N-D1 fragment (Zhang *et al.*, 2000 ▶) were used to compute anomalous difference maps that provided the locations of 50 of the 57 Se atoms within the asymmetric unit (three protomers with 19 methionine residues per protomer in the asymmetric unit). Although the resolution of the MAD phase data sets was limited (∼5.5 Å), the experimental phases improved phase-combined electron-density maps such that backbone tracing of the D2 domain became possible (DeLaBarre & Brunger, 2003 ▶).

During the backbone tracing of the D2 domain, polyserine helices were placed into the electron density in regions that had ‘sausage-like’ character. The positions of the polyserine helices were refined by rigid-body minimization. These initial helices confirmed the expected structural similarity between the D1 and D2 domains. Using the homology to the D1 domain as a guide, the polyserine model for the D2 domain was further extended to produce models for the β-sheets and some information on loop connectivity. Using the known Se positions of the 19 methionine residues in each p97/VCP protomer greatly facilitated tracing, although in retrospect the Se positions were not sufficient to uniquely assign the backbone positions of the corresponding methionine residues, resulting in many register shifts of the polypeptide backbone (Davies *et al.*, 2008 ▶).

With most of the p97/VCP structure and the substructure of the anomalous scatterers determined, we performed iterative model building and refinement to improve both the p97/VCP atomic model and the parameters of the selenomethionine substructure. Phase probability distributions of the current p97/VCP atomic model were computed. Next, these model phase probability distributions were used as ‘prior’ distributions to assist the maximum-likelihood refinement of the selenium substructure. New experimental phase probability distributions (without the prior distributions) were then computed from the refined phasing model and used to assist the maximum-likelihood refinement of the entire p97/VCP atomic model using the MLHL target function (Pannu *et al.*, 1998 ▶; Adams *et al.*, 1999 ▶). This loop was repeated to convergence of the standard phasing statistics and electron-density map quality. NCS restraints and secondary-structure restraints (using tight backbone hydrogen-bond distance restraints for α-­helices and β-sheets) were applied during refinement. *B*-­factor sharpening to phase-combined electron-density maps yielded maps which enabled tentative assignment for ∼30% of the side chains in the D2 domain. The phase-combined electron-density maps also allowed identification of the bound nucleotide as ADP·AlF_*x*_ in the D2 domain and ADP in the D1 domain. Alternating rounds of positional and group *B*-factor refinement and manual rebuilding resulted in a final model with reasonable *R* and *R*_free_ values. Subsequently, crystal structures of p97/VCP in the ADP and AMP-PNP nucleotide states were determined and refined (DeLaBarre & Brunger, 2005 ▶). Tight NCS restraints were used independently for all subdomains (N, D1α, D1α/β, D2α, D2α/β), omitting the inter-domain linkers. Experimental phase information was used in all refinements. The resulting structures exhibited reasonable statistics (*R* values in the range 30–40% with good covalent geometry) but had many outliers in the Ramachandran plots. However, the electron-density maps did not provide any clues to how to further improve the model.

At the time, four full-length p97 structures were available in four hydrolysis states: ATP (from PDB entry 1ypw; 3.5 Å resolution), ADP·AlF_*x*_ (from PDB entry 1yq0; 4.5 Å resolution), ADP (from PDB entry 1yqi; 4.25 Å resolution) and, independently, apo (PDB code 1r7r; 3.6 Å resolution; Huyton *et al.*, 2003 ▶). These structures suggested that p97/VCP primarily undergoes motion at two stages of the nucleotide-hydrolysis cycle: between the ATP and ADP·AlF_*x*_ states and between the ADP and apo states (DeLaBarre & Brunger, 2005 ▶). Three regions undergo order–disorder transitions during the hydrolysis cycle: the D2α domain, the D1–D2 linker region and the sensor-2 region of the D2 domain. Nucleotide-induced domain motions from the D2 domain are transmitted *via* the D1–D2 linker region to the D1α domain (DeLaBarre & Brunger, 2003 ▶). The D1α domain makes multiple contacts with the N domain and serves to regulate the motion of the N domain. Significant deviations from sixfold symmetry were observed for some of the subunits (DeLaBarre & Brunger, 2005 ▶).

### 3.2. Re-refinement using high-resolution structures for all domains

**Figure 1:**
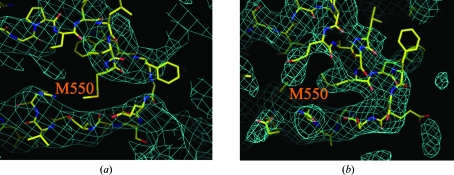
Improvement of model fit and electron-density map quality for the re-refined p97/VCP structure in complex with ADP·AlF_*x*_. A loop in D2, residues 530–570, is shown. Phase-combined σ_A_-weighted 2*F*_o_ − *F*_c_ maps (blue mesh) were calculated using the ADP·AlF_*x*_ diffraction data to 4.4 Å resolution and either (*a*) the final model (sticks) and refinement parameters from the original model (PDB code 1yqo) and (*b*) the re-refined model (PDB code 3cf1). The refinement used the MLHL target function with the bulk-solvent correction as implemented in *CNS* v.1.2. *B*-sharpening was applied (*B*_sharp_ = −120 Å^2^). Note that although the backbone position of Met550 is shifted in the new models, the relative position of the sulfur is preserved and is in agreement with experimental selenomethionine data. Upon re-refinement the free *R* value improved by 5.2% (from 33.8% to 28.6%) and the *R* value improved by 3.9% (from 26.8% to 22.9%).

Subsequent to the publication of the original p97/VCP structures, a high-resolution structure of the D2 domain (PDB code 3cf0; 3.0 Å resolution with 14-fold noncrystallographic symmetry) became available (Davies *et al.*, 2008 ▶). This new structure, in conjunction with that of the N-D1 fragment ND1 (PDB code 1e32; 2.9 Å resolution), allowed re-refinement against the low-resolution diffraction data for the full-length p97/VCP crystal structures. Although only a few residues were added to the models, the revised full-length models showed significant improvement in secondary-structure geometry, *R* values and electron-density maps (Fig. 1 ▶). The free *R* values fell by as much as 5% compared with the original structure refinements, indicating that there is information in the diffraction data even at ∼4 Å resolution that objectively assesses the quality of the model.

The refinement protocol was very similar to that used for the original p97/VCP refinements, including the use of experimental phase information during refinement, NCS restraints and group *B*-factor refinement. However, no secondary-structure restraints were required to maintain good local secondary-structure definition. The availability of the high-resolution structure of the D2 domain was essential to obtain these improved structures since the electron-density maps derived from the original models were poorly defined in several regions (Fig. 1 ▶). In fact, for the re-refined structures, the improvements upon *B*-factor sharpening were more pronounced than for the original p97/VCP structures, more clearly defining side chains (Fig. 1 ▶). The regions that showed the most improvement in density were those that were less well defined, such as the D1–D2 linker region. Inspection of the *B*-sharpened electron-density maps did not reveal any regions that were significantly degraded relative to the nonsharpened maps, so *B*-sharpening was maintained for model building.

The overall fold of full-length p97/VCP was unchanged upon re-refinement, although there were differences in detail, including many register shifts, which produced overall root-mean-square deviations (r.m.s.d.s) of 3.2 and 3.4 Å for C^α^ atoms relative to the original structures for the ADP and ADP·AlF_*x*_ states, respectively. Many of the more significant differences between re-refined and previous models resided in the D2 nucleotide-binding site. In the original models of the site, conserved chemical features were placed in significantly different positions in each nucleotide state. For instance, the adenine rings in previous models occupied substantially different portions of the binding pocket, resulting in variable contacts between the ring and the surrounding protein and discrepant positions for the surrounding residues amongst the states. In the re-refined structures the conformation of the nucleotide and the contacts made with the adenine ring remained relatively unchanged across the nucleotide states.

The AMPPNP-bound p97/VCP crystal diffracted to the highest resolution (3.5 Å) of the full-length crystals, but even with fourfold NCS averaging the electron-density maps for this state were poorly defined in certain areas, the most significant of which was the D2 α-helical domain as we had previously noticed in the original structure determination (DeLaBarre & Brunger, 2005 ▶). As a consequence, much of the D2 α-helical subdomain was still missing in the re-refined model, although the re-refinement helped to clarify several areas of poorer definition, such as the D1–D2 linker region.

As had already been found in the original models, the ADP and ADP·AlF_*x*_ crystal structures exhibit significant asymmetry; that is, they are each composed of three non-identical protomers per asymmetric unit. In the original ADP·AlF_*x*_ structure the AlF_*x*_ moiety was fully occupied in only one of the three D2 protomers, but upon re-refinement using identical symmetry restraints the nucleotide was found to be present in all copies with occupancies near one. Although the nucleotide-state and binding-site configuration were found to be the same from one protomer to the next, the relative arrangement of domains differed among the protomers in both crystals.

Our re-refinements of the p97/VCP structures confirmed the large conformational differences between nucleotide states and the asymmetry between protomers, in particular in the ADP state, that had previously been observed (DeLaBarre & Brunger, 2005 ▶). However, the re-refinement significantly improved the quality of the models, allowing more detailed analysis of the observed conformational changes between nucleotide states (Davies *et al.*, 2008 ▶), which allowed the elucidation of probable mechanisms of differences in hydrolysis rates between D1 and D2, as well as the mechanism of transmission of nucleotide-state information between sub­units.

## 4. Conclusions

Our experience with the p97/VCP low-resolution crystal structures shows that if high-resolution models of most portions of the structure are available, they should be used as starting points for refinement. In the absence of such information, *de novo* model building is highly problematic at low resolution; our original tracing of the D2 domain resulted in many register errors even though some SeMet positions and homology were used as a guide during the model building. Compared with our original structures, the free *R* values, agreement with electron-density maps and Ramachandran statistics significantly improved upon the re-refinements starting from high-resolution structures. Thus, even low-resolution diffraction data contain information to objectively assess the quality of the model. These examples also show that atom-model refinement using the latest versions of refinement programs (*CNS* and *Phenix*) is possible and desirable even at low resolution. However, inspection of electron-density maps and current model-building tools fail to indicate how to improve the model (see, for example, Fig. 1 ▶). Thus, there is a need for the development of new computational tools to achieve the maximum possible and most accurate information from low-resolution diffraction data using all available prior information (*e.g.* from homology models) even if high-resolution structures are unavailable.

## Figures and Tables

**Table 1:** Considerations for low-resolution refinements

1	Use high-resolution structures as starting points for the refinement if available
2	Make the most of the diffraction data by including weak reflections using the resolution-dependence of σ_A_ as a guide to determine the effective resolution limit
3	If possible, use experimental phases (*e.g.* SAD or MAD experiment) in refinement and electron-density maps since experimental phases add both model-independent information and increase the effective number of observables *versus* parameters
4	If available, use the location of SeMet positions to guide the model building
5	Use optimal bulk-solvent model and scaling methods for low resolution (Brunger, 2007 ▶)
6	Exploit geometric redundancies (NCS and/or multiple crystal forms) to improve experimental phases for the computation of electron-density maps and to constrain or restrain the model
7	Restrict or restrain refinement to minimum necessary degrees of freedom
8	Try *B*-sharpening electron-density maps
